# Effect of Imperfections Due to Material Heterogeneity on the Offset of Polysilicon MEMS Structures

**DOI:** 10.3390/s19153256

**Published:** 2019-07-24

**Authors:** Aldo Ghisi, Stefano Mariani

**Affiliations:** Department of Civil and Environmental Engineering, Politecnico di Milano, Piazza Leonardo Da Vinci 32, 20133 Milano, Italy

**Keywords:** polysilicon MEMS, uncertainty estimation, statistical volume element, zero offset

## Abstract

Monte Carlo analyses on statistical volume elements allow quantifying the effect of polycrystalline morphology, in terms of grain topology and orientation, on the scattering of the elastic properties of polysilicon springs. The results are synthesized through statistical (lognormal) distributions depending on grain size and morphology: such statistical distributions are an accurate and manageable alternative to numerically-burdensome analyses. Together with this quantification of material property uncertainties, the effect of the scattering of the over-etch on the stiffness of the supporting springs can also be accounted for, by subdividing them into domains wherein statistical fluctuations are assumed not to exist. The effectiveness of the proposed stochastic approach is checked with the problem of the quantification of the offset from the designed configuration, due to the residual stresses, for a statically-indeterminate MEMS structure made of heterogeneous (polycrystalline) material.

## 1. Introduction

Because of the microfabrication process, a polysilicon MEMS structure is never found at the exact designed place, but after the production steps, a shift from the rest position is often observed [1,2]. The difference can be so small to be inconsequential for the working conditions, but in particular cases, it can represent a significant disturbance. Since the phenomenon depends on the actual geometry and material involved, it is intrinsically of a stochastic nature. To deal with the consequences of these uncertainties quantitatively, a statistical approach should be therefore followed, by introducing a probabilistic distribution for the relevant variables involved in the manufacturing process.

This variable initial offset creates a series of uncertainties that require workarounds or specific electronics to account for them. The most common remedy is of the type exemplified by [3], where an initial offset due to process mismatch (called “zero-g offset” or “intrinsic sensor offset”), generating a variation in the initial capacitance of the sensor up to 10% and 20% for a three-axis accelerometer, is treated through a compensation circuit. Another calibration (or auto-calibration) procedure was shown in [4], to account for the varying gap geometry of MEMS featuring comb drives due to the fabrication process. More importantly, the mentioned uncertainties affect the stiffness of critical components, spreading it around the designed value: a plethora of methods, a good review of which can be found in [5], has been invented to overcome this problem. Stiffness is in fact a key parameter for defining relevant design variables, for example the resonance frequency: e.g. in [6] it was discussed how, because of the uncertainty on the geometry due to the manufacturing process, it is a challenging objective to obtain a tight sense-drive frequency separation (e.g. 2%) for an MEMS gyroscope, since the stiffness cannot be controlled finely enough.

When one considers the previous examples, it is understandable that uncertainties affecting the initial positions are however related to the ones responsible for the variability from the target status (i.e. not due to drifts or noise) during the MEMS working conditions: they are in fact both generated by the same sources during the manufacturing process. For example, in [7] a list of sensitive parameters, namely key geometrical dimensions (varying about 6–10%) or material properties (varying about 10%) (as taken from [8]) was used to explore how the uncertainty propagates in a mathematical model describing the working conditions of an MEMS energy harvester: it is easy to recognize that the same quantities would affect an offset from the designed configuration as well.

It would be therefore a useful insight to quantify the role of these uncertainties in an effective and manageable way, before moving to the working conditions, where other issues could be involved. Moreover, the corrections necessary to overcome the initial offset can become themselves too costly to be economically convenient, as shown in [3].

Going more into the details, the sources of these uncertainties can be (i) the so-called over-etch generated from the sequence of deposition, masking alignment, photolithography, and the deep reaction-ion etching process [9,10,11] and (ii) the effect of the material heterogeneity due to the polycrystalline morphology [12,13,14]: the former affects the geometry layout, while the latter influences the effective material properties. Both the causes tend to be neglected or hastily (and roughly) estimated, when the dimensions of critical MEMS components are significantly larger than a characteristic length of the microstructure, such as the average silicon grain size. However, both of these causes become relevant as far the miniaturization proceeds and the aforementioned critical dimensions shrink.

In the past, the authors devised a numerical approach to foresee the mechanical behavior of a polycrystalline ensemble by carefully representing, through an artificial reconstruction, the network of the grain and grain boundaries [15,16,17,18]. The approach is typically used to carry out a homogenization procedure and to establish the conditions necessary to build a Representative Volume Element (RVE) [19,20], but it can also be adopted to study Statistical Volume Elements (SVEs), i.e. to construct a statistical set accounting for all the desired uncertainties (such as the grain topology and the size and orientations of the elastic axis) whenever the characteristics at the micro scale become non-negligible for the mechanical quantities. This condition exactly arises when the miniaturization is put to the current technological limit and the silicon grain size becomes on the order of magnitude of the minimum dimensions of the characteristic structural parts, like the width of the slender suspension beams.

From the perspective of the quantification of the uncertainties due to material heterogeneity, in this work we aim to extract the information necessary to create quantitatively-informed stochastic analytical distributions of the elastic properties, such as the apparent Young’s moduli *E* and *G* and Poisson’s ratio ν, from numerical simulations of the SVEs. The latter represent only a realization of the stochastic variables involved in the definition of the mechanical properties at the upper, device-level scale, different from the more commonly-encountered RVEs that would represent the effective properties in a deterministic sense. However, the knowledge of the statistics of the aforementioned elastic quantities allows rigorously quantifying their scattering around a mean value, and therefore to foresee the mechanical uncertainty transferred to the structural behavior, e.g. the suspension spring’s stiffness.

With regard to the geometrical uncertainty due to over-etch fluctuations, different from the commonly-adopted simplification of a constant value for a single device, in this work we explore the effect of a scattering from the mentioned constant value along the supporting beams. In this case, an analytical distribution is (a priori) assumed, and the contribution of variable over-etch on the beam moment of inertia is considered in addition to the previously-mentioned material-generated uncertainty.

The main purpose of our approach is to overcome the computational burden of the polycrystalline morphology analysis, thanks to the analytical distributions, which are therefore good to devise an engineering tool useful for design.

In the following Section 2, an exemplary MEMS configuration possibly leading to an offset is described. Then, the procedure exploiting (i) Monte Carlo (MC) analyses of SVEs aiming to define the elastic mechanical properties of homogenized polysilicon, (ii) the extraction of the mentioned analytical stochastic distributions from the numerical data, and (iii) their employment to build the (statistically-informed) stiffnesses of critical MEMS details is carefully detailed in Section 3. This reasoning is adopted to solve a typical offset problem in Section 4, where a discussion of the outcome is also carried out.

## 2. Sensitivity to Imperfections: A Simple Model for MEMS Offset

**Figure 1:**
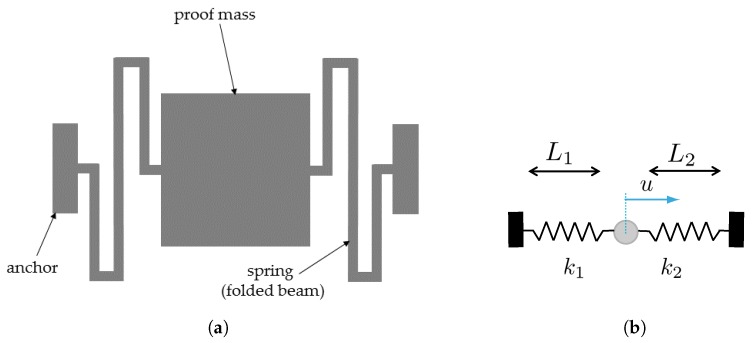
(**a**) Example and (**b**) structural scheme of single-axis inertial MEMS devices, featuring a proof mass anchored to the die via two springs in series.

The effect of mechanical and geometrical uncertainties at the microscale can be clearly observed in the case of statically-indeterminate movable structures. As a benchmark example, inspired by the geometry of single-axis inertial MEMS devices working as shown in Figure 1 (see also [21,22,23]), we focus on a simplified scheme, where a proof mass is connected to the substrate through a couple of polycrystalline silicon beams or springs in series. In this case, even if the target design is to have the two stiffnesses $k_{1}$ and $k_{2}$ equal, the randomly-varying grain morphology, the over-etch defects [9], and the residual stresses arising from the manufacturing process induce an offset *u* away from the rest position.

Whatever the geometry of the two springs is, the said imperfections cause instead the stiffness values $k_{1}$ and $k_{2}$ to differ. The resulting offset *u* that can detrimentally affect the performance indices of the device linked, e.g. to capacitive readout, often proves negligible, but it may become relevant when the dimensions of critical structural details (like e.g. the in-plane spring width) become comparable to the average silicon grain size or to the microfabrication tolerances related to the etching stage. By assuming the proof mass to be a rigid body, in order to compute *u* we assume two sources that induce the (either positive or negative) elongation of the springs: an inelastic deformation $\epsilon_{r}$ linked to the residual stress in the polysilicon film; an elastic deformation $\epsilon_{e}$ induced by the constraints at the anchor points that prevent any motion at the end points. The latter effect can be formally represented by a force *F* acting on both the springs in series. Due to the said constraints at the anchors, the compatibility equation for this statically-indeterminate system is given by:

(1) $$F\;\left(\frac{1}{k_{1}}+\frac{1}{k_{2}}\right)+\epsilon_{r}\;\left(L_{1}+L_{2}\right)=0.$$

Within the proposed frame, the sources $\epsilon_{r}$ and *F* are assumed without any dependence on the out-of-plane direction; possible effects of residual stress gradients are therefore disregarded to simplify the analysis, with a focus only on the in-plane motion of the proof mass. Moreover, the lengths $L_{1}$ and $L_{2}$ of the springs are meant in a very general sense as kinds of effective values, in order to allow also for folded geometries like the one depicted in Figure 1a. Solving Equation (1) for *F*, we end up with:

(2) $$F=-\frac{L_{1}+L_{2}}{\frac{1}{k_{1}}+\frac{1}{k_{2}}}\;\epsilon_{r}$$

and the offset *u* thus reads:

(3) $$u=\frac{\frac{k_{1}}{k_{2}}L_{1}-L_{2}}{1+\frac{k_{1}}{k_{2}}}\;\epsilon_{r}.$$

For the sake of simplicity, we now assume $L_{1}=L_{2}=L$. To avoid in the analysis any dependence on $\epsilon_{r}$, which, as stated, represents an inelastic effect of the residual stresses and stands as a kind of algorithmic, model-based parameter, Equations (2) and (3) are solved for it, and the results are given next in terms of the ratio u/F, according to:

(4) $$\frac{u}{F}=\frac{1-\frac{k_{1}}{k_{2}}}{2\;k_{1}}$$

which depends on the stiffnesses of the springs only. Equation (4) shows that, if the two stiffnesses are equal (i.e. $k_{1}=k_{2}$), no offset is induced. When, instead, microscale scattering leads to different stiffness values, the offset shows up. To account for such discrepancy between the two values of the spring stiffness, we assume that the scattering induces the values $k_{1}=k+\Delta k_{1}$ and $k_{2}=k+\Delta k_{2}$, with $\Delta k_{1}\neq \Delta k_{2}$ (where *k* can be assumed as the target reference value). Equation (4) is then modified as:

(5) $$\frac{u}{F}=\frac{1-\frac{k+\Delta k_{1}}{k+\Delta k_{2}}}{2\left(k+\Delta k_{1}\right)}=\frac{\Delta k_{2}-\Delta k_{1}}{2\;\left(k+\Delta k_{1}\right)\;\left(k+\Delta k_{2}\right)}.$$

It is noted that a difference in the stiffness values is not sufficient to get an offset: the force *F* arising from the aforementioned residual stress distribution is also required to trigger the proof mass displacement. This through-the-thickness distribution of the residual stresses is actually a whole problem on its own [24]: though the simple model (4) can be adopted to estimate them via MC techniques like that suggested in [25], provided that measurements are available for a number of statistically-representative devices, in this work we do not aim to address this issue, but instead a quantification of the uncertainty effects linked to the stiffness of structural components. As mentioned in the Introduction, we handle two sources of the scattering of the stiffness values: the heterogeneity of the polycrystalline material and the geometrical imperfections due to the manufacturing process. Both micromechanical sources are specifically discussed in Section 3.

## 3. Characterization of the Uncertainties at the Microscale

To quantify the uncertainties in the spring stiffness, the two sources linked to the polycrystalline morphology and to the etch defects are dealt with separately. In Section 3.1, an MC procedure is adopted to determine the homogenized elastic properties of the polysilicon film, in terms of microstructure-affected values of Young’s modulus *E*, shear modulus *G*, and Poisson’s ratio ν. Since we considered polycrystalline silicon with a columnar structure, the material was treated as transversely isotropic at the device scale. The homogenized elastic properties obtained with the MC procedure at the micro-scale refer to the plane parallel to the substrate, so that the out-of-plane direction coincides with the grain columns. Due to the FCC crystal lattice of silicon, Young’s moduli obtained from the homogenization along two orthogonal directions are always equal, and deviations from isotropy prove small. Next, in Section 3.2, these scattered overall properties are used jointly with the statistics of over-etch to provide an estimate of the scattering in the overall spring stiffness according to beam bending theory.

### 3.1. Apparent Elastic Properties of Polysilicon Films

Let us start by allowing for the uncertainties linked to the microstructure of the polysilicon film, namely the morphology of the polycrystal. As already stated, we focus here on the in-plane motion of the structure; therefore, a two-dimensional geometry was considered. To assess the impact of the microstructure on the dispersion of the results, an MC procedure was adopted; within such a stochastic procedure, each realization or sample of the polysilicon film had the shape of a square of side-length *ℓ*. This length-scale *ℓ* was assumed to be linked to the characteristic size of the already mentioned critical details of the geometry of the movable structure. In the simple geometry of Figure 1, as the proof mass was assumed rigid, *ℓ* had to be driven by the springs’ features: out of their length and in-plane width, the latter obviously drives scattering at the material level and is therefore considered in what follows. As far as the length was instead concerned, the way to handle it will be detailed in Section 3.2.

**Figure 2:**
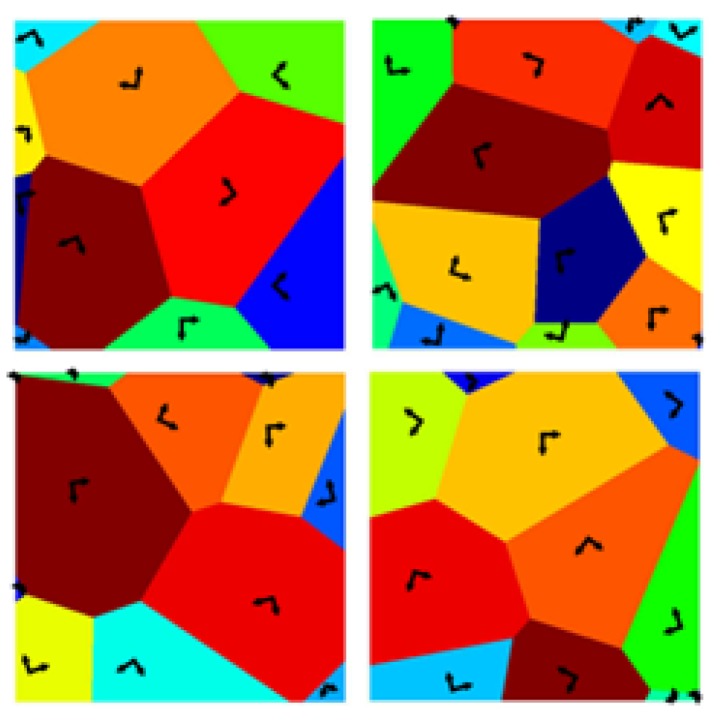
The 2×2μm${}^{2}$ SVE examples of the in-plane polycrystalline morphology adopted in the MC analysis.

The microstructure inside each square sample was obtained via a (regularized) artificial Voronoi tessellation, by imposing an average grain size g=1μm [17] measured as the average distance between the centroids of the grains in the tessellation. Due to the columnar morphology of the polysilicon film [26], with a texture almost aligned with the out-of-plane axis for all the grains, the randomness of crystal lattice was assumed to hold in-plane only [19]. Because of the small ℓ/g ratio, the length-scale separation assumption of homogenization did not hold true, and relevant asymptotic bounds on the elastic properties of the RVEs did not actually cover the full range of values of microstructure-affected apparent elastic properties: outliers therefore happened to fall out of the bilateral limits, as observed in [14]. The sampling of values out of the range provided by asymptotic, analytical bounds was assumed to undermine the assumption of representativeness, and the results thus had to be interpreted in a statistical context. To understand the above reasoning, in Figure 2 a few examples of the microstructural samples are shown, reporting also the orientation of the in-plane axes of elasticity for each grain.

In the numerical, Finite Element (FE) procedure feeding the MC analysis, the whole sample was meshed with quadratic triangular elements featuring a characteristic size of 125 nm, to assure accuracy in the results. This element size was already proven in [17,27] to lead to mesh-independent results, in terms of the homogenized elastic property of the polycrystalline samples. To decrease the computational burden of the simulations, especially in view of the number of samples to be handled in each MC analysis, some authors proposed the use of (grain) boundary element formulations; see e.g. [28,29,30,31], wherein only the grain boundary network has to be discretized to reduce the dimensionality of the problem.

The reasons for relying on a numerical strategy with numerical simulations to feed an MC analysis were already discussed in [27]. If one is interested in the reference or mean values of the mechanical properties of the polysilicon film, asymptotic approaches will suffice; if the scattering induced by micromechanical features is instead the main focus of the analysis, a proper statistical description of all the stochastic variables and a proper sampling out of the relevant statistical distributions are indeed necessary. The aforementioned need is strongly enhanced in the considered case of ℓ/g values on the order of 2–3. To this end an SVE, as handled in the analysis, was therefore defined as follows [32,33]: it is a domain whose dimension is smaller than a conventional RVE, but larger than the characteristic length scale (i.e. the grain size), so that the elastic properties obtained from the homogenization procedure are not to be considered as the effective ones to be adopted for a homogeneous deterministic device-level analysis, but as representative of the actual microstructure randomness. These elastic properties have to be considered as the “apparent” ones, according to the definition provided in [32]: i.e. the apparent Young’s modulus obtained from each SVE is simply a realization of a random variable, which accounts for the relevant microstructure, and it should not be used in a deterministic analysis at the device-level scale.

For the MC analysis, two different sets of SVEs were handled with ℓ=2,3μm. These two values were selected in accordance with our previous studies [12,13,14]: to emphasize the microstructural effects, ℓ/g has to be minimized in compliance with the microfabrication constraints. This rationale justified the value ℓ=2μm, whereas ℓ=3μm was selected on the basis of the dimension of beams in the structure described in [34].

The apparent elastic moduli of each sample were obtained according to the procedure discussed in [17]. A bilateral bounding scheme was adopted by imposing either uniform stress or uniform strain Boundary Conditions (BCs) all over the boundary of the SVE. Periodic BCs were not adopted, since a periodic microstructure can never be observed in real polysilicon films. For each type of BCs, three analyses were run, inducing in turn only one non-zero component of the macroscopic in-plane stress or strain; the results of these three analyses then allowed estimating the microstructure-affected values of *E*, *G*, and ν. The latter values turned out to depend on the film morphology due to the local intensification of the stress and strain fields caused by the misorientation of adjacent grains. Additional details, which are beyond the scope of this work, can be found in [17]; see also [27].

**Figure 3:**
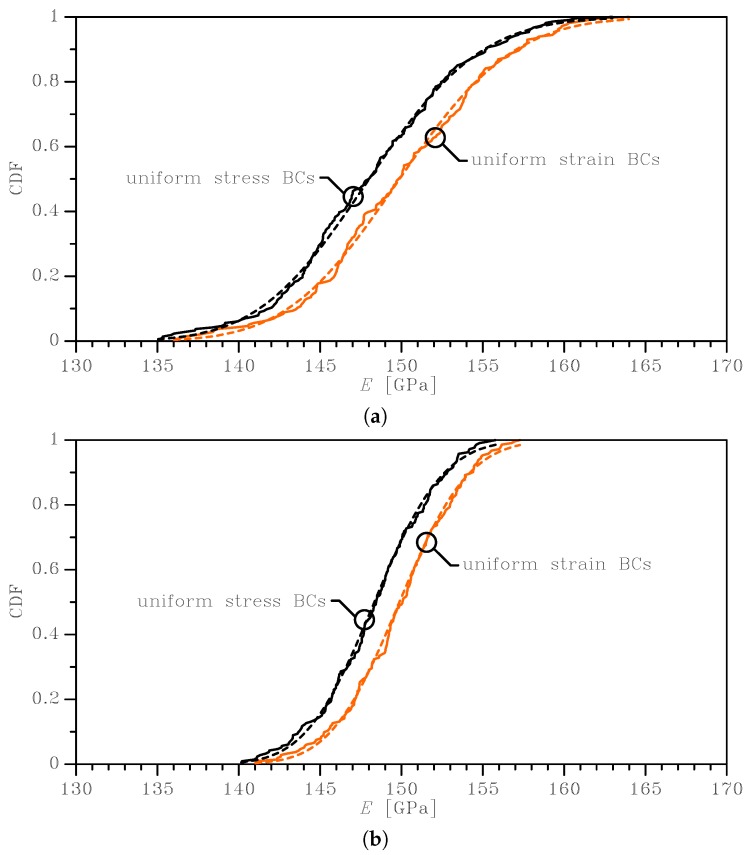
Effects of SVE size and BCs on the cumulative distribution function of the homogenized Young’s modulus: (**a**) 2 × 2 μm${}^{2}$ SVE and (**b**) 3 × 3 μm${}^{2}$ SVE. Continuous lines represent the results of MC analyses, whereas dashed lines are the relevant lognormal interpolants.

**Figure 4:**
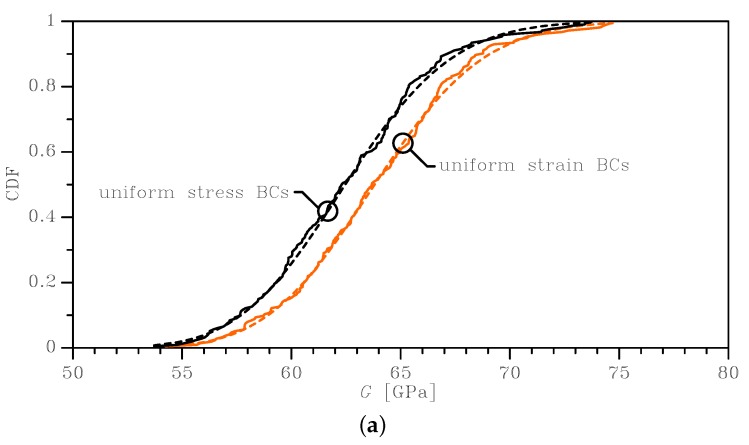

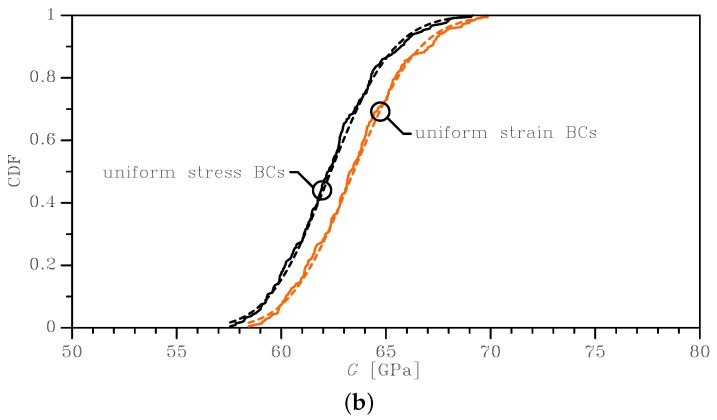
Effects of SVE size and BCs on the cumulative distribution function of the homogenized shear modulus *G*: (**a**) 2 × 2 μm${}^{2}$ SVE and (**b**) 3 × 3 μm${}^{2}$ SVE. Continuous lines represent the results of MC analyses, whereas dashed lines are the relevant lognormal interpolants.

**Figure 5:**
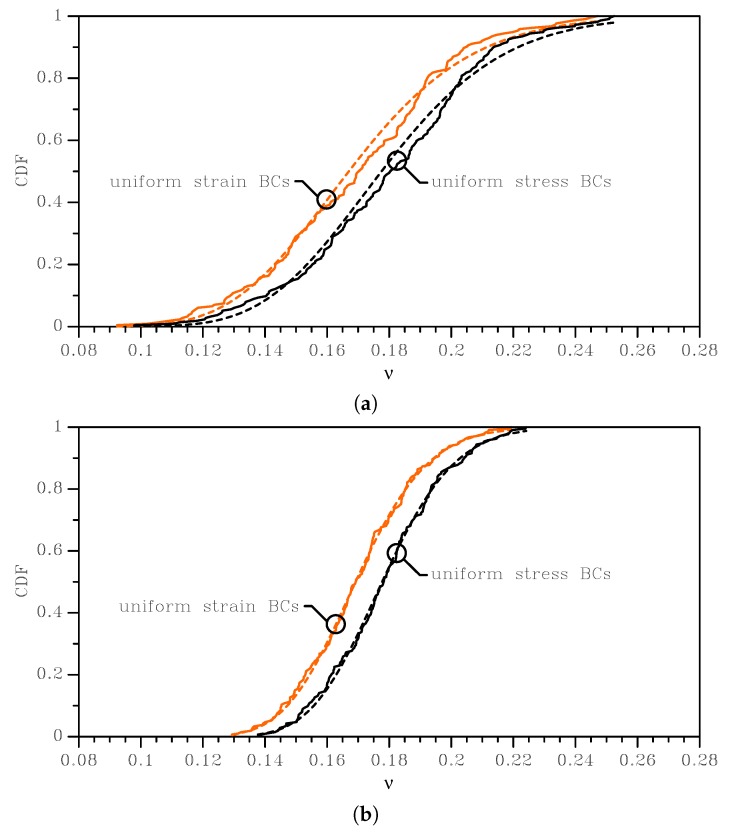
Effects of SVE size and BCs on the cumulative distribution function of the homogenized Poisson’s ratio ν: (**a**) 2 × 2 μm${}^{2}$ SVE and (**b**) 3 × 3 μm${}^{2}$ SVE. Continuous lines represent the results of MC analyses, whereas dashed lines are the relevant lognormal interpolants.

**Figure 6:**
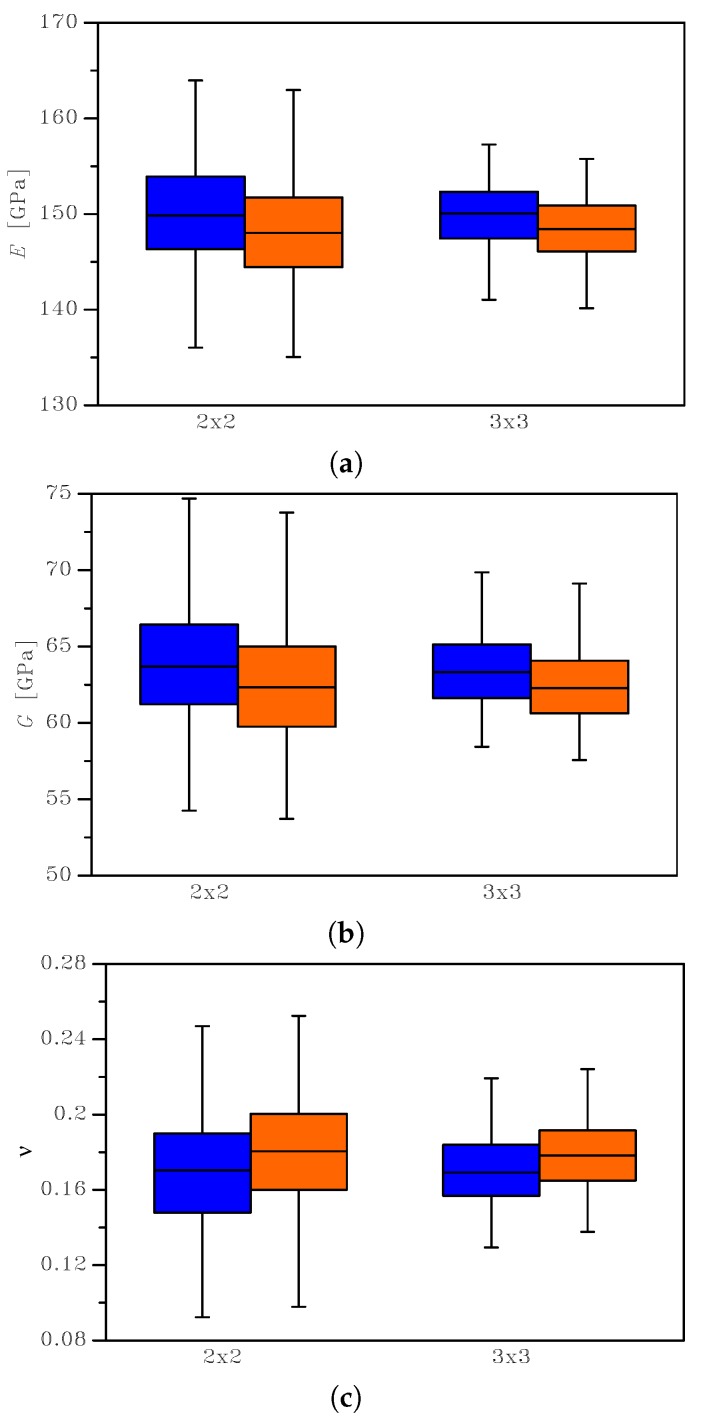
Box-whisker plots of the homogenized elastic properties of the SVE: effects of the BCs (blue: uniform strain BCs; orange: uniform stress BCs) on the scattering of (**a**) Young’s modulus, (**b**) shear modulus, and (**c**) Poisson’s ratio.

The outcomes of this statistical analysis are reported with continuous lines in Figure 3, Figure 4 and Figure 5, in terms of the Cumulative Distribution Functions (CDFs) of *E*, *G*, and ν, as obtained with the two types of BCs and for the two SVE sizes. As is known, the uniform strain BCs lead to an upper bound on the actual value of *E* and *G* and a lower bound on the actual value of ν; vice versa, the uniform stress BCs provide a lower bound for *E* and *G* and an upper bound for the actual ν. The plots allow also to compare easily the 2×2μm${}^{2}$ and the 3×3μm${}^{2}$ cases and to quantify the effect of a smaller device-level size *ℓ* with respect to the average grain size: it marginally affects the mean values of the elastic moduli, while the scattering around them is strongly modified. The same results are also collectively reported in Figure 6 as box-whisker plots, to show in a compact way the mean values and the dispersion around them. No limits were assumed to exclude possible outliers in the data, so the full range of values of the results was included in the plots.

The mean value of the apparent Young’s modulus was slightly dependent on the BCs (as expected, see [32] and see Table 1): for the 2 × 2 μm${}^{2}$ case, it amounted to *E* = 149.98 GPa for uniform strain BCs, while it read *E* = 148.11 GPa for uniform stress BCs. It was almost negligibly affected (on the order of 1%) by the dimension of the SVE (*ℓ* = 2 or 3 μm) for the assumed grain size g=1μm. The results showed instead a larger influence of the SVE size on the standard deviation, as qualitatively shown in Figure 3 by the less steep CDF curve for the 2 × 2 μm${}^{2}$ case with respect to the 3 × 3 μm${}^{2}$ case. Quantitatively, the standard deviation for the uniform strain BCs increased from 3.34 GPa to 5.47 GPa when the SVE size decreased from ℓ=3μm to ℓ=2μm: the correspondent coefficients of variations (i.e. the ratio between the standard deviation and the mean value) were around 2.2% and 3.5%, respectively. We can interpret this larger scattering as the quantification of the effect of the grain morphology on the statistical distribution of *E*, as the grain size becomes comparable with the SVE size.

Similar considerations can be made for the apparent shear modulus *G*; see Table 2: the scattering between the mean values was small (on the order of 2%) when the BCs and/or the SVE size were varied; the scattering became more significant if the standard deviation was considered, as evidenced by the values of the coefficients of variations, ranging from 3.9% (3 × 3 μm${}^{2}$) to 6.2% (2 × 2 μm${}^{2}$).

For the apparent Poisson’s ratio (see Table 3), the mean values did not change significantly with the BCs, but they varied with the reduction of the SVE size of about 9%. The corresponding standard deviation instead showed a more meaningful change, varying from about 10% for the 3 × 3 μm${}^{2}$ case to about 17% for the 2 × 2 μm${}^{2}$ case.

**Figure 7:**
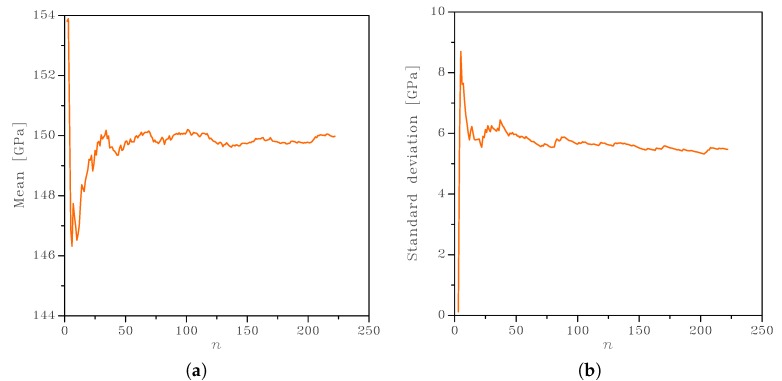

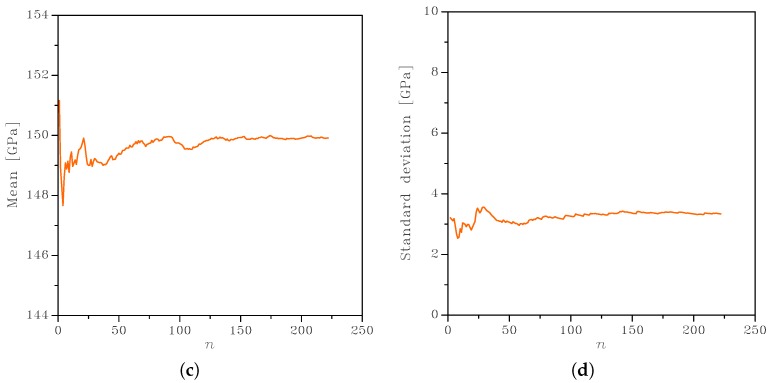
Effect of the number *n* of samples in the Monte Carlo analysis on the convergence of the (**a**,**c**) mean value and (**b**,**d**) standard deviation of in-plane Young’s modulus *E*, for uniform strain BCs. Top: 2 × 2 μm${}^{2}$ SVE. Bottom: 3 × 3 μm${}^{2}$ SVE.

As in any MC analysis, results relevant to a sufficiently high number of samples have to be collected to guarantee the convergence in the statistics of the apparent properties. With the focus on the homogenized Young’s modulus of the polycrystalline film, some exemplary results are collected in Figure 7 for the 2 × 2 μm${}^{2}$ SVE (top row), in terms of the convergence of the mean value and of the standard deviation with an increasing number *n* of samples. Besides some small fluctuations, visible only because of the reduced scale of the graphs, outcomes tended to converge and became stable even before *n* = 100. For the 3 × 3 μm${}^{2}$ SVE shown in Figure 7 (bottom row), the convergence was attained even faster, as expected for a larger SVE. Such an evolution of the graphs was not directly related to some specific SVE morphologies, since they remained unchanged (within the limits of the statistical representation) by shuffling the order of the SVEs handled in the MC analysis. Similar results were observed for any elastic property of the film, and for any SVE size; the only slight difference at varying SVE size was the different value at convergence for the standard deviation.

**Figure 8:**
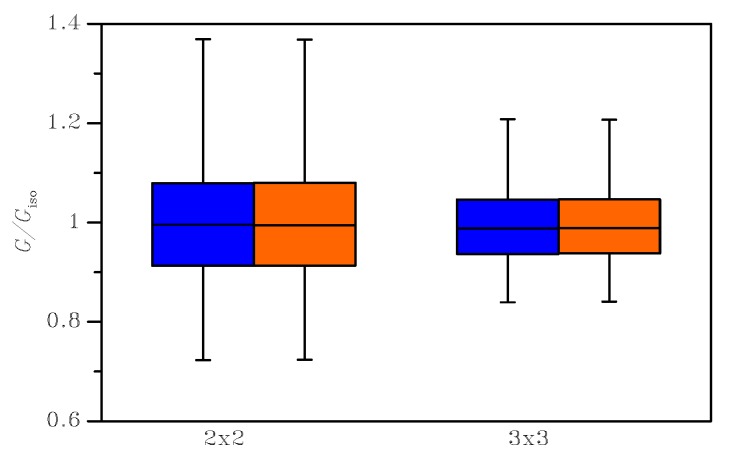
Box-whisker plot for the ratio between $G/G_{\mathrm{iso}}$ (blue: uniform strain BCs; orange: uniform stress BCs).

A further interesting feature of the mechanical response of the polysilicon film was its in-plane degree of anisotropy. In Figure 8, box-whisker plots are reported, again at varying BCs and SVE sizes, for the ratio $G/G_{\mathrm{iso}}$, where $G_{\mathrm{iso}}=E/\left(2\;\left(1+\nu \right)\right)$ would be the shear modulus of a perfectly in-plane isotropic material, whose Young’s modulus *E* and Poisson’s ratio ν were those obtained by means of the same homogenization procedure. This measure was already adopted in [17] to assess the material-dependent isotropy of microstructured materials and, to some extent, the representativeness of the SVE size. Since silicon, due to its FCC crystal structure, is a moderately-anisotropic material, the results related to aggregates of randomly-oriented grains are supposed to be characterized by a ratio $G_{\mathrm{iso}}=E/\left(2\;\left(1+\nu \right)\right)$ tightly bonded around isotropy, i.e. around $G/G_{\mathrm{iso}}=1$. This ratio in the case of single crystal silicon, which has a low degree of anisotropy, turns out to be 1.57; it can be therefore appreciated that, in the case of a polycrystalline material, the ratio tends towards a unitary value, even for small SVEs as the ones considered in this work (see Figure 2).

To deal with the analysis of the imperfection sensitivity at the spring scale, the numerical statistical distributions were fitted with analytical lognormal CDFs (represented by the dashed lines in the graphs of Figure 3, Figure 4 and Figure 5):

(6) $$C\left(\xi \right)=\;\frac{1}{2}+\frac{1}{2}\;\mathrm{erf}\left(\frac{\ln\xi -\mu }{\sqrt{2}\omega }\right)$$

where μ is the mean value and ω is the standard deviation of the natural logarithm of the random variable ξ (i.e. alternatively *E*, *G*, or ν); erf(·) is the normalized Gaussian function (also known as the “error function”). These distributions statistically satisfied the constraint for *E* and *G* to be always positive, with no artificial procedures necessary to avoid handling negative elastic moduli in the calculation of the overall spring stiffness; see also [35]. Moreover, an analytical, but SVE-informed CDF had the advantage of the ease of handling in further theoretical developments, as we will show for the spring stiffness in Section 3.2.

In Table 1, Table 2 and Table 3, the discussed mean and the standard deviation values obtained with the homogenization procedure are shown, together with the parameters μ and ω of Equation (6) as identified through a nonlinear fitting. These latter parameters made the analytical lognormal distribution ready to be used for the estimate of the stiffnesses of MEMS springs whose polysilicon morphology showed a grain size on the order of g=1μm. The quality of the adopted fitting via the lognormal distributions was quantified by the very small standard deviations shown in the tables for the parameters μ and for ω in all the considered cases: for the mean and standard deviation values corresponding to *E*, *G*, and ν, the scattering was even smaller and not affecting the least significant digit there reported.

The choice of the probability distribution for the fitting of the results of the MC analysis was a relevant decision. A lognormal probability distribution was assumed for each elastic modulus, but we assumed independence among *E*, *G*, and ν. This latter hypothesis does not prove always correct: in this way, in fact, one neglects the influence of the microstructure on the correlation among them. However, since in this work, we used the outcomes of the stochastic analysis to compute the stiffness of slender beams depending on the Young’s modulus only, the hypothesis did not detrimentally affect the results. This would not be the case for more complex inferences, i.e. in the case of the stiffness evaluation for a shorter beam, where also *G* and shear deformations play a role. In these cases, a multivariate probability distribution should be considered, and the correlations between the variables should be evaluated, e.g. through marginal distributions. Assuming that the dependency structure of the multivariate probability distribution would be static, i.e. the stochastic process is stationary, copulae could be used to evaluate the said marginal probability distributions. For time-evolving stochastic processes, other approaches such as spectral analysis would be necessary. Both copulae and spectral analysis are out of the scope of this paper; interested readers can find information on them, e.g. in [36,37], respectively.

### 3.2. Overall Spring Stiffness Calculation

**Figure 9:**
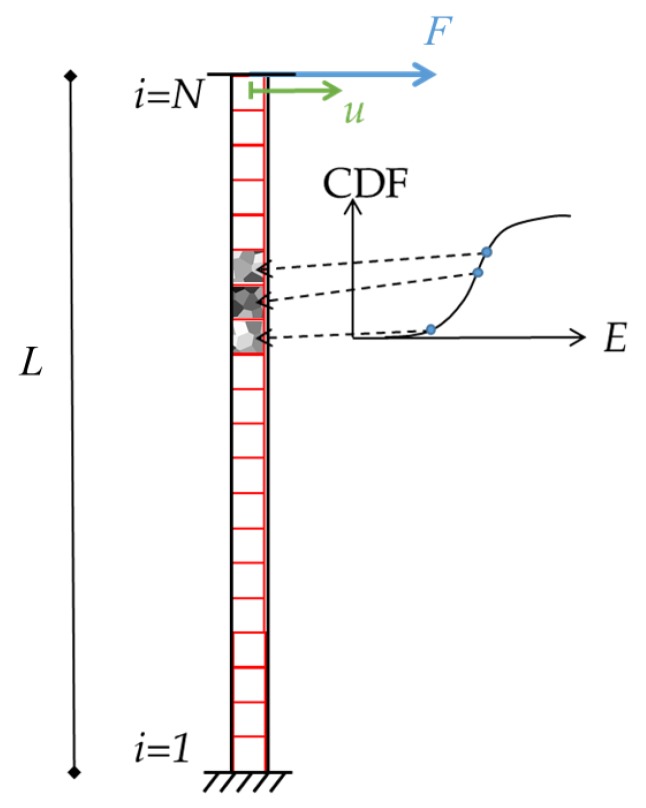
Scheme adopted for the calculation of the scattered spring stiffness with the spring itself subdivided into *N* subsets, each one with a Young’s modulus sampled from the relevant size-dependent CDF and a moment of inertia dependent on the over-etch.

For the suspension spring configuration shown in Figure 9, the overall stiffness entering into play in Equation (5) with the relevant scattering can be computed as follows. Results can be easily generalized to the case of folded beams, since the stiffness scales inversely proportional to the number of folds. Stiffness values reported for homogeneous beams (see [38]) cannot be adopted in the present statistical analysis due the local heterogeneity of the material that governs the problem.

We assumed the beam to be subdivided into *N* disjoint subdomains, like in domain decomposition procedures [39,40,41]: within each subdomain, Young’s modulus *E* was assumed constant; as far as the moment of inertia was instead concerned, we assumed that, for the sake of simplicity, its value varied for each subdomain according to a Gaussian distribution accounting for the scattering of the over-etch. The mentioned value of *E* was sampled out of the lognormal fitting of the statistical distributions obtained from the analysis at the polycrystalline material level. In the analysis to follow, we will consider springs characterized by high values of the ratio between length and in-plane width, namely very slender or thin geometries: Young’s modulus is then the only parameter affecting the solution, according to the mathematical procedure described in the Appendix. In the case of thick springs, shear stresses gain importance in the solution, and also the distribution of *G* should be accounted for; in such a case, the correlation between the distributions of *E* and *G* has to be also allowed for in the analysis.

The adopted procedure is schematically reported in Figure 9: a beam has a fixed constraint at one end, where displacements and rotations are prevented, and a slider at the opposite end, where the motion is in the direction perpendicular to the beam axis only, without any rotation. The latter end is supposed to be the link to the proof mass, which moves as a rigid body. By means of the principle of virtual work and allowing for beam slenderness to neglect shear strains, the force *F* and the corresponding displacement *u* can be related to provide the ratio F/u as the beam stiffness *k*.

The outcome of this reasoning, described in detail in Appendix A, depends on the number of subdivisions *N* along the beam length, and it turns out to provide the spring stiffness as:

(7) $$k=\frac{F}{u}=\frac{1}{L^{3}}{\left(\sum_{i=1}^{N}\frac{\psi_{i}}{E_{i}\;I_{i}}\right)}^{-1}$$

where *L* is the beam length, *i* is the index running over the subsets handled, $I_{i}$ is the moment of inertia of the *i*th subset, $E_{i}$ is the apparent Young’s modulus at the *i*th subset, and $\psi_{i}$ is a corrective factor dependent on the placement of the *i*th subset and defined in Equation (A10). Since the overall stiffness is a function of the random variables (E,I), then it is a random variable as well.

When the moment of inertia is assumed constant along the beam, the expression of the stiffness simplifies to:

(8) $$k=\frac{F}{u}=\frac{I}{L^{3}}\left(\frac{1}{\sum_{i=1}^{N}\frac{\psi_{i}}{E_{i}}}\right).$$

## 4. Evaluation of the Offset at Rest

### 4.1. Effect of Material Uncertainties Only

In this section, the CDFs of the offset of the proof mass from its rest position are reported as obtained from Equation (5), in which *u* was taken as unknown, while the stiffnesses were calculated with two alternative choices: (i) from the lognormal CDFs as described in Section 3.2 or (ii) directly from FE simulations of the two-dimensional L×w (e.g. 200 × 2 μm${}^{2}$) beam with the geometry shown in Figure 9. In these latter FE analyses, a random silicon grain morphology was considered for the whole domain: it is worthwhile to emphasize that the computational burden of these analyses was very high, since hundreds of grains were involved. To characterize the statistics of the stiffness via FE analyses of the whole beam, 200 simulations were carried out by varying in each case the whole silicon grain morphology.

As for the CDF of *F* in Equation (5) resulting from the production process, we considered the experimental data shown in [42] measured in a 0.3 μm-thick polysilicon layer: a residual strain constant through the thickness $\epsilon_{r}=360\pm 5\;\cdot 10^{-6}$ was reported. In absence of further information, we assumed a Gaussian distribution for $\epsilon_{r}$ with the mentioned mean and standard deviation, and we simply computed $F=\tilde{E}\;\epsilon_{r}\;A$, $\tilde{E}=150$ GPa being a reference Young’s modulus and *A* the beam cross-section area. Therefore, the distribution of *F* was Gaussian as well.

**Figure 10:**
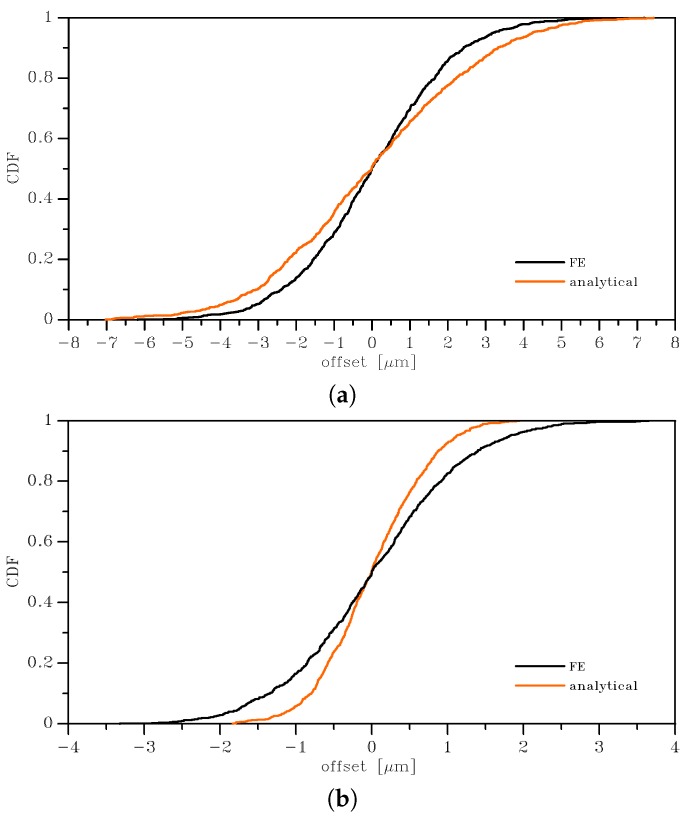
Offset from the rest position for length × SVE size cases: (**a**) 200 × 2 μm${}^{2}$ and (**b**) 200 × 3 μm${}^{2}$.

In Figure 10, the offset CDFs derived from the stiffnesses obtained through the sampling of the apparent Young’s modulus from the (analytical) lognormal distributions are shown with orange lines, while the CDFs derived from the stiffnesses obtained from the FE simulations are instead reported with black lines. An almost zero offset mean value was common to the two cases, so the main difference between the two solutions was related to the variance of *E* and, therefore, of *k*. Looking at the results, we can conclude that the semi-analytical approach, which was far less expensive than the FE one, correctly represented the information obtained from the FE analyses. Around the zero mean value, the offset became positive or negative depending on the difference between the stiffnesses of the right and left springs (see Figure 1). It is noted that the offset was generated by the scattering of the values of the spring stiffnesses around the mean; therefore, it depended on the standard deviations of the CDFs, and not on the mean values. Any stochastic method providing an offset estimate should then address also the quantification of the variance of the random variables associated with the elastic properties, not only their mean values.

**Figure 11:**
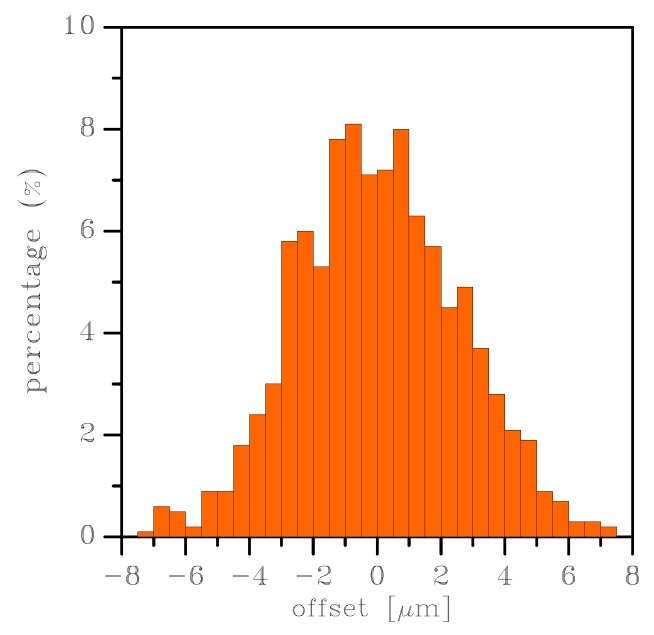
Histogram of the offset from the rest position for the 200 × 2 μm${}^{2}$ case (uniform strain BCs).

An alternative representation is provided in Figure 11 for the 200 μm × 2 μm case. The histogram represents the percentage of a given realization with a certain value of offset from the rest position. Though not further investigated in this work, it can be seen that the distribution of the offset was not unimodal.

### 4.2. Effect of Variable Over-Etch and Material Property Uncertainties

**Figure 12:**
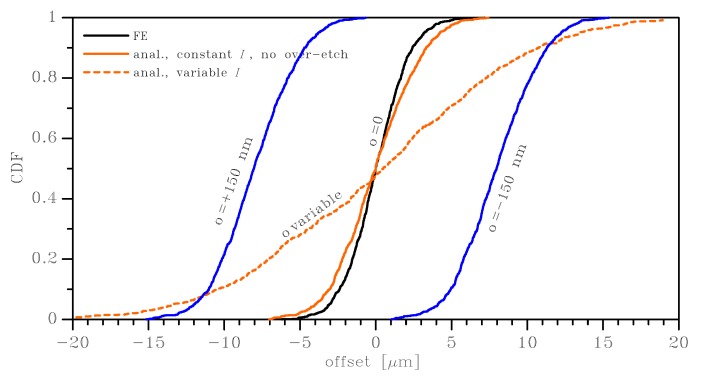
Effect on the offset of the variable (over-etch affected) moment of inertia *I* and Young’s modulus along the beam length (200 × 2 μm${}^{2}$ case, uniform strain BCs). Blue lines refer to fixed values for the over-etch equal to ±150 nm.

In Figure 12, in addition to the effect of a variable Young’s modulus according to the rationale described above, we took into account the scattering of the geometry due to the over-etch variation along the beam length. We assumed that the over-etch *o* along the beam was distributed as a Gaussian variable with a zero mean value and a standard deviation equal to 50 nm; for the *i*th domain along the beam, the corresponding moment of inertia was thus computed as $I_{i}=t\;{\left(w-2\;o_{i}\right)}^{3}/12$, where $o_{i}$ is the value of the over-etch sampled out of the aforementioned Gaussian distribution. o=0 corresponds to the commonly-assumed over-etch reference value in the engineering practice: a constant value for a single MEMS structure or even for MEMS distributed at different positions on a wafer. The graphs in Figure 12 include, as a reference, the previous curves for FE and analytical results related to the effects due to the scattering of Young’s modulus only: the scattering due to a variable moment of inertia was shown to be far larger, as evidenced by the lower slope of the dashed orange curve and by the corresponding standard deviation, which increased from 2.5–8.2 μm, while the mean value was about zero in all cases. Two additional curves, in blue lines, were also added to the graph: they represent the scattering of the offset, due to the uncertainty on the material only, when the two springs in Figure 1 differed in width exactly by a value of 150 nm, which is representative of the 95% confidence interval from the target during the manufacturing process [12,43]. It can be seen that most of the offsets fell within the range dictated by the two blue curves.

## 5. Conclusions

We proposed a stochastic method to evaluate the offset from the rest position for a statically-indeterminate MEMS typology, featuring a proof mass supported by two opposite springs. When the polysilicon production process was pushed to the current limit, the uncertainties about the stiffnesses of critical MEMS parts, such as nominally-identical supporting beams, generated an offset from the designed position when a residual stress distribution was also present inside the structure. Both material heterogeneity and fluctuations in critical geometry dimensions, i.e. the beam width, could be responsible for such an offset. For the first cause, we addressed the definition of the elastic properties accounting for the polycrystalline morphology by an artificially-reconstructed Voronoi tessellation in terms of statistically-representative elements, whose dimensions were equal to a characteristic dimension of the spring, namely their width, and were comparable with the silicon grain. For the second cause, we considered the effect of the over-etch on the moment of inertia of the MEMS supporting beams.

The classical homogenization procedure, under the hypothesis of statistical independence of the variables, provided a stochastic insight into the relevant mechanical properties, in particular the elastic moduli and Poisson’s ratio, through numerical cumulated distribution functions that were subsequently approximated by an analytical, lognormal distribution. The stiffness of each beam supporting the moving mass was then estimated via the analytical lognormal distribution according to a simple reasoning based on the principle of virtual work applied to an exemplary beam.

By considering a statistically-indeterminate beam-moving mass-beam chain, we assumed as known the internal force due to residual stresses: it generated an offset from the rest position even in the presence of a stiffness varying only according to the polycrystalline material. A variable over-etch along the supporting beams, however, was responsible for a higher offset than the one induced by uncertainties on the material properties only. An important conclusion was that the evaluation of the offset depended on the correct characterization of the variance of Young’s modulus and the moment of inertia, not on their mean value.

The approach shown here for a very simple class of structures could be extended to other statically-indeterminate MEMS configurations, provided that they are modeled as beam systems, in case the heterogeneity of the polysilicon is a matter of concern.

## Figures and Tables

**Table 1:** Young’s modulus *E*: apparent values of the mean and standard deviation obtained with the MC analysis and parameters for the lognormal CDFs in Equation (6).

Case	Mean (GPa)	Std Deviation (GPa)	μ	ω
2 × 2 μm${}^{2}$, uniform strain BCs	149.98	5.47	5.010 ± 0.003	0.037 ± 0.002
2 × 2 μm${}^{2}$, uniform stress BCs	148.11	5.40	4.997 ± 0.003	0.037 ± 0.002
3 × 3 μm${}^{2}$, uniform strain BCs	149.91	3.34	5.010 ± 0.002	0.022 ± 0.001
3 × 3 μm${}^{2}$, uniform stress BCs	148.37	3.32	4.997 ± 0.002	0.023 ± 0.001

**Table 2:** Shear modulus *G*: apparent values of the mean and standard deviation obtained with the MC analysis and parameters for the lognormal CDFs in Equation (6).

Case	Mean (GPa)	Std Deviation (GPa)	μ	ω
2×2 μm${}^{2}$, uniform strain BCs	63.93	3.98	4.156 ± 0.004	0.062 ± 0.003
2×2 μm${}^{2}$, uniform stress BCs	62.58	3.91	4.134 ± 0.004	0.062 ± 0.003
3×3 μm${}^{2}$, uniform strain BCs	63.51	2.46	4.150 ± 0.003	0.038 ± 0.002
3×3 μm${}^{2}$, uniform stress BCs	62.41	2.38	4.133 ± 0.003	0.038 ± 0.002

**Table 3:** Poisson’s ratio ν: apparent values of the mean and standard deviation obtained with the MC analysis and parameters for the lognormal CDFs in Equation (6).

Case	Mean	Std Deviation	μ	ω
2×2 μm${}^{2}$, uniform strain BCs	0.180	0.030	−1.790 ∓ 0.013	0.184 ± 0.009
2×2 μm${}^{2}$, uniform stress BCs	0.170	0.030	−1.728 ∓ 0.012	0.173 ± 0.008
3×3 μm${}^{2}$, uniform strain BCs	0.179	0.018	−1.777 ∓ 0.008	0.108 ± 0.005
3×3 μm${}^{2}$, uniform stress BCs	0.170	0.018	−1.727 ∓ 0.007	0.103 ± 0.005

## Abbreviations

The following abbreviations are used in this manuscript:

BC	Boundary Condition
CDF	Cumulative Distribution Function
FE	Finite Element
MC	Monte Carlo
RVE	Representative Volume Element
SVE	Statistical Volume Element

## Appendix A

To quantify the stiffness of the supporting springs in the reference configuration of Figure 9, we relied upon the principle of virtual work for slender beams with a Bernoulli–Euler kinematics. When a force *F* acts perpendicularly to the longitudinal axis of the beam, it causes an internal bending moment M. At equilibrium, the external work $W_{e}$ equals the internal work $W_{i}$, which read:

(A1) $$W_{e}=F_{u}$$

(A2) $$\begin{array}{cc} W_{i} & =\int_{0}^{L}M\left(\zeta \right)\;\chi \left(\zeta \right)\;d\zeta \end{array}$$

where *u* is the virtual displacement of force *F* in the same direction (hence, perpendicular to the beam axis), χ is the virtual curvature of the beam axis, and ζ is the axis aligned with the longitudinal direction of the spring.

For a statically-indeterminate structure, if both the elastic modulus *E* and the moment of inertia *I* are constant all along the beam axis, the solution to $W_{e}=W_{i}$ can be found in any textbook of structural mechanics. In the present case, where both *E* and *I* can vary stochastically as functions of ζ, M(ζ) and χ(ζ) need to be determined. The variation of the bending moment along the spring axis reads M=M−Fζ, where *M* is the value of the top end of the beam, as induced by the local constraint that prevents any in-plane rotation. Hence, in compliance with the mentioned constraint, we have:

(A3) $$0=\int_{0}^{L}\frac{M-F\zeta }{E(\zeta )\;I(\zeta )}\;d\zeta .$$

The integral was solved numerically, by subdividing the beam into *N* disjoint domains of the same longitudinal length Δζ=L/N; within each of them, we assumed that the flexural rigidity was locally constant, i.e. stochastic fluctuations were locally neglected. Accordingly, we arrive at:

(A4) $$\sum_{i=1}^{N}\frac{1}{E_{i}\;I_{i}}\;\int_{\zeta_{i}}^{\zeta_{i}+\Delta \zeta }\left(M-F\zeta \right)\;d\zeta =0$$

where $E_{i}$ and $I_{i}$ are the Young’s modulus and the moment of inertia for the *i*th domain, respectively. By solving for *M*, we obtain:

(A5) $$M=\frac{\sum_{i=1}^{N}\frac{\Delta \zeta +2\zeta_{i}}{2\;E_{i}\;I_{i}}}{\sum_{i=1}^{N}\frac{1}{E_{i}\;I_{i}}}\;F.$$

In compliance with the adopted subdivision of the beam, we also have $\zeta_{i}=\left(i-1\right)\Delta \zeta$, and Equation (A5) becomes:

(A6) $$M=\frac{F\;L}{2}\;\gamma$$

where:

(A7) $$\gamma =\frac{\sum_{i=1}^{N}\frac{2i-1}{N\;(E_{i}\;I_{i})}}{\sum_{i=1}^{N}\frac{1}{E_{i}\;I_{i}}}$$

is a factor that allows for the non-uniform value of the flexural rigidity of the beam and modifies the value M=FL/2 relevant to the uniform beam case.

Now that the internal moments are fully given as a function of the external force *F*, we can solve $W_{e}=W_{i}$ for the displacement *u* to obtain:

(A8) $$u=\int_{0}^{L}\frac{F{\left(\frac{L}{2}\gamma -\zeta \right)}^{2}}{E(\zeta )I(\zeta )}\;d\zeta$$

being $\chi =\frac{M}{E(\zeta )\;I(\zeta )}$. With the help of the same subdivision of the beam into *N* domains adopted to compute *M*, we finally obtain Equation (7), here rewritten for convenience:

(A9) $$k=\frac{F}{u}=\frac{1}{L^{3}}{\left(\sum_{i=1}^{N}\frac{\psi_{i}}{E_{i}\;I_{i}}\right)}^{-1}$$

where:

(A10) $$\psi_{i}={\left(\frac{i-1}{N}-\frac{\gamma }{2}\right)}^{2}\frac{1}{N}+\left(\frac{i-1}{N}-\frac{\gamma }{2}\right)\frac{1}{N^{2}}+\frac{1}{3N^{3}}.$$
